# Modelling Competing Endogenous RNA Networks

**DOI:** 10.1371/journal.pone.0066609

**Published:** 2013-06-26

**Authors:** Carla Bosia, Andrea Pagnani, Riccardo Zecchina

**Affiliations:** 1 Human Genetics Foundation (HuGeF), Torino, Italy; 2 Physics Department and Center for Computational Sciences, Politecnico Torino, Torino, Italy; Memorial Sloan Kettering Cancer Center, United States of America

## Abstract

MicroRNAs (miRNAs) are small RNA molecules, about 22 nucleotide long, which post-transcriptionally regulate their target messenger RNAs (mRNAs). They accomplish key roles in gene regulatory networks, ranging from signaling pathways to tissue morphogenesis, and their aberrant behavior is often associated with the development of various diseases. Recently it has been experimentally shown that the way miRNAs interact with their targets can be described in terms of a titration mechanism. From a theoretical point of view titration mechanisms are characterized by threshold effect at near-equimolarity of the different chemical species, hypersensitivity of the system around the threshold, and cross-talk among targets. The latter characteristic has been lately identified as competing endogenous RNA (ceRNA) effect to mark those indirect interactions among targets of a common pool of miRNAs they are in competition for. Here we propose a stochastic model to analyze the equilibrium and out-of-equilibrium properties of a network of 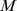 miRNAs interacting with 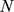 mRNA targets. In particular we are able to describe in detail the peculiar equilibrium and non-equilibrium phenomena that the system displays in proximity to the threshold: (i) maximal cross-talk and correlation between targets, (ii) robustness of ceRNA effect with respect to the model's parameters and in particular to the catalyticity of the miRNA-mRNA interaction, and (iii) anomalous response-time to external perturbations.

## Introduction

A recently discovered molecular mechanism [1], lately named Competing Endogenous RNA (ceRNA) effect [2], [3], points out the importance of indirect interactions among transcript RNAs in competition for the same pool of microRNAs (miRNAs). MiRNAs are small – about 22 nucleotide long – non-coding RNAs which post-transcriptionally interact with their targets in a sequence dependent manner. In their mature stage, miRNAs get included in a RNA-induced silencing complex (RISC) and, eventually, thanks to a 6–8 nucleotide long seed region, bind specifically the miRNA regulatory elements (MREs) in the 3′UTR of their target mRNAs. The effective miRNA/mRNA interaction turns out to be very complex and still poorly understood. Depending on (i) the degree of complementarity of the seed region with the target, (ii) the interaction of miRNAs with Argonaute preoteins which induces functional domains ( *e.g.* anchor, seed, central, 3′ supplementary, and tail regions) [4] on the miRNA sequence, miRNAs can either cleave the transcripts or downregulate their translation: in either case the net effect is a reduced amount of mRNAs or proteins. MiRNAs are known to regulate a multitude of different processes ranging from differentiation to neural plasticity, and their misfunctioning is often associated with the development of diseases [5], [6].

In a nutshell the idea behind the ceRNA effect boils down to the simple observation that, while interacting with a target mRNA, a single miRNA cannot act on other targets. Mature miRNAs (*i.e.* miRNAs loaded in RISC) are thus the limiting factor in a system of potentially interacting target mRNAs. If for example gene A, which shares one miRNA with gene B, is up-regulated the common miRNAs will tend to bind preferentially to mRNA A due to its increased concentration. Consequently, mRNA of gene B will be less repressed resulting in a subsequent increased concentration [1]–[3], [7], [8]. Other studies have independently provided further evidences for miRNA mediated trans-regulatory mRNA effects [9], [10]. Since each miRNA can have several targets, a complex indirect interaction network among different targets emerges, where nodes are mRNA transcripts and there is a link between two nodes if they have at least one miRNA in common. Then, the highest the number of common miRNAs or MREs, the strongest the link. Such crosstalk effect has been observed in bacteria where the role of miRNAs is played by small RNAs (sRNAs) and it is due to a titrative interaction among sRNAs and targets [11]. Depending on the number of sRNA binding elements crosstalk among sRNA targets can then be prioritized and selective [11], [12].

Interaction via titration mechanisms entails a threshold-like behavior between the two interacting molecules, where the threshold position is determined by the relative amount of them [11], [13]–[16]. This means that as long as the concentration of one of these two molecules is below the threshold almost all of them are bound in complexes with the second ones and their free amount is very low. Increasing their concentration beyond the threshold results in an increased amount of free molecules, while the others will be in turn almost all bound in complexes. Moreover, systems of molecules interacting in a titrative fashion also show a hypersensitivity in proximity to the threshold to changes in the molecule production rates [13], [14]. In particular controlled conditions it has been shown that it is right near the threshold, where sensitivity is maximal, that crosstalk among sRNA targets is maximal too [11].

Remarkably, Mukherji and co-workers [17] recently observed a threshold-like effect also in miRNA target expression in single cells. Moreover, in line with studies in bacteria [11], [15] and with earlier works on protein-protein interaction [13], [14], they tested a mathematical deterministic model of molecular titration to describe their results and found it in good agreement with experimental observations. Such results strengthen the idea that behind the ceRNA effect there is a miRNA-target titration mechanism.

Motivated by [17] and [2], [3] and by results obtained in experiments with bacteria [11], [12], [15], in this paper we extend previous models to the case of a general network of 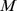 miRNAs titratively interacting with 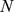 target mRNAs (ceRNAs) and analyze it from a stochastic point of view. So far analytical predictions from models for titrative interactions did not go beyond the mean-field limit [11], [15], [17], [18] or were limited to the case of small circuits because of the nonlinearities involved [13]. However, (i) stochasticity plays a central role in gene expression mostly when numbers of molecules involved are modest [19]–[21] and (ii) small circuits are usually embedded in more complex networks so that induced interactions might be relevant. Since potential crosstalk among miRNA targets is effective right in proximity to the threshold, where free chemical species (*i.e.* not bound in complexes) are present in small numbers, it is necessary a stochastic analysis of the system.

Here we show that, despite the complexity and the intrinsic non-linearity of the system, a shrewd use of the moment generating function approach plus a simple Gaussian approximation are enough to obtain analytical expressions for noise and Pearson's correlation coefficients for all the molecular species considered in a generic network.

As a preliminary result we describe, at the level of the independent molecular species approximation (*viz.* mean-field), the onset of a threshold-like behavior typical of titration mechanism [11], [13]–[16], which has been specifically investigated in [17], [18] in the case of a miRNA-mediated mRNA interaction, and discuss the possible mechanism leading to a specificity of the interactions.

Secondly, for the first time, we derive analytical results beyond the independent molecular species approximation which allows for the characterization of profiles for means, noise and Pearson's correlation coefficients, comparing them with numerical simulations. Interestingly, we found that in proximity to the threshold both noise (in terms of Fano factor and coefficient of variation) and correlation profiles among the different molecular species show a maximum. Even if the noise increases, ceRNAs and miRNAs fluctuate in a highly correlated manner. Titration-like interactions could thus be an adequate mechanism to affects system's homeostasis, possibly supporting the idea of miRNAs as key players in conferring robustness to the system [22]–[24].

Among the different parameters characterizing miRNA-mRNA interactions, the degree of catalyticity – *i.e.* the fraction of miRNA molecules that are recycled after the interaction with their target – is among the most disputed yet less understood ones: [25], [26] support an almost completely catalytic interaction (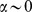), while at the opposite range [27]–[29] support an almost completely stoichiometric interaction (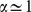). Finally, intermediate values of catalyticity are indeed supported by a recent work [30]. Here we show that ceRNA effect is robust with respect to this parameter too. In the limiting case of a completely catalytic interaction (*i.e.* 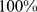 of the miRNA is recycled) a threshold-behavior is still observed as an intrinsically out-of-equilibrium phenomenon: the location of the threshold turns out to be a monotonously increasing function of time such that, at equilibrium (long-time limit), no threshold behavior is observed.

An out-of-equilibrium characteristic of the system predicted by the model is the response time of a ceRNA embedded in a network after a single factor perturbation. Again, in proximity to the threshold, we observe peculiar trends: upon switching on or off another ceRNA in the network the response times show a maximum and a minimum respectively, and the qualitative profiles are independent of the number of ceRNAs in competition.

Finally we conclude proposing a series of specific experiments aiming at validating both qualitatively and quantitatively the model's predictions, and briefly describing how ceRNA interaction turns out to be stable in presence of more complex network topologies such as feedback and feedforward loops.

## Results

### Definition of a network of interaction miRNAs-ceRNAs

**Figure 1:**
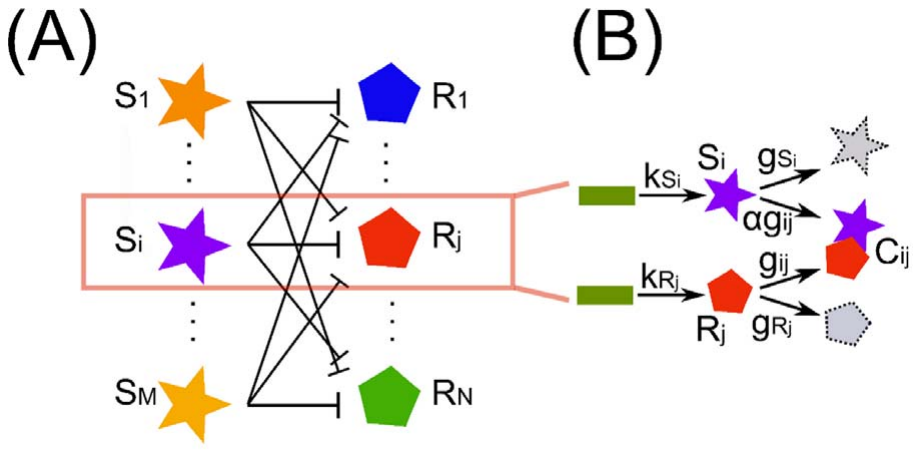
Representation of a generic miRNA-target interaction network. (A) Simplified picture of a miRNA-ceRNA interaction network. (B) For each miRNA (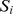) and ceRNA (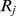) present in the network we consider the main steps of transcription (rates 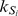 and 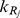 respectively) and degradation (rates 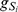 and 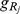 respectively) plus a titrative interaction between miRNA and ceRNA. miRNA and ceRNA can therefore form a complex 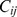 with effective association rate 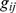. The parameter 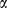 (the catalyticity parameter) tells which is the probability a miRNA is recycled after having interacted with one of its targets.

The network we are interested in describing is schematically depicted in Figure 1A, where 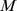 different free mature miRNAs (colored stars) can interact with 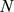 different free target mRNAs (colored pentagons). miRNAs and target mRNAs interact via a titration-like mechanism [17]. As a first approximation we can think the mRNAs as irreversibly lost due to the miRNAs actions (miRNA-target association rate much greater than dissociation rate) while the miRNAs can eventually be recycled. Such approximation is supported by recent results on the estimate of the miRNA-target complex dissociation rate [4]. Figure 1B shows a cartoon of such mechanism in which two different DNA molecules (green rectangles) are transcribed with rates 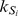 and 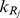 to become miRNA 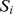 and mRNA 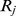 respectively. Eventually 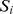 and 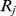 either degrade (broken gray stars and pentagons) with rates 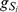 and 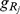 or interact binding in a complex 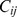 via an effective association rate 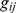.

The effective association rate 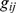 should be thought as a combination of association, dissociation and degradation rates of the miRNA-mRNA complex 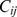 (see Supporting Information (File S1) for more details). Once in the complex the mRNA 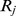 cannot be translated or utilized anymore. The parameter 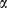 (with 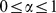) is a measure of the catalyticity of the miRNA, that is the ability the miRNA has to be available again once having interacted with its target. Thus, 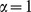 means that for each mRNA 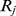 bound in a complex 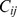 there is also one miRNA 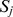 sequestered (and no more able to interact with its other targets) while 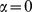 implies that mRNA 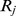 effective degradation is increased by 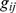 but this does not have any effect on the miRNA 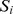.

### Mean field approximation: threshold behavior and cross-talk

**Figure 2:**
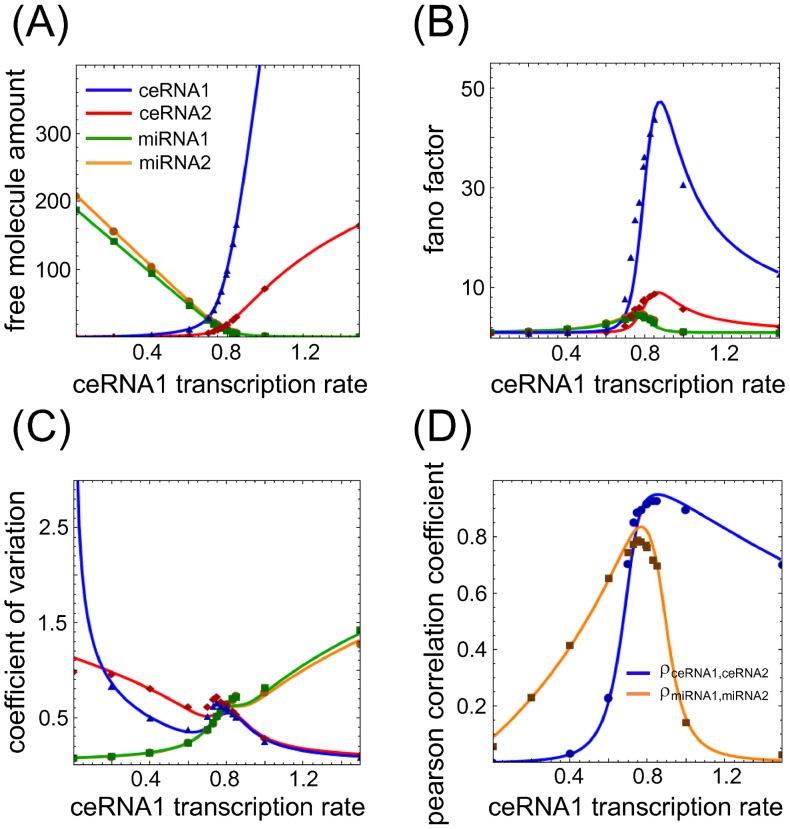
Threshold, noise and Pearson's coefficients varying ceRNA transcription rate. (A–C) Steady state value for means, Fano factors and coefficients of variation for each free molecular species in a system of two miRNAs (miRNA1 and miRNA2, green and orange lines respectively) interacting with two ceRNAs (ceRNA1 and ceRNA2, blue and red lines respectively) varying the concentration of ceRNA1. In proximity to the threshold the system shows hypersensitivity to changes in the control parameter (ceRNA1 transcription rate), captured by a maximum in the Fano factors (panel B). For the same values of ceRNA1 transcription rate, the local maximum in the coefficients of variation (panel C) is the fingerprint of bimodal distributions in the number of molecules for each molecular species. (D) Pearson's coefficients between the two miRNAs (orange line) and the two ceRNAs (blue line). The two lines show a maximum in proximity to the ceRNA1 transcriptiom rate threshold value, meaning that there is a region of parameters where the fluctuations in the number of ceRNAs or miRNAs are highly correlated. Lines are the results of Gaussian approximation while symbols are Gillespie's simulations. For panels B,C the line color-code is the same as in panel A.

**Figure 3:**
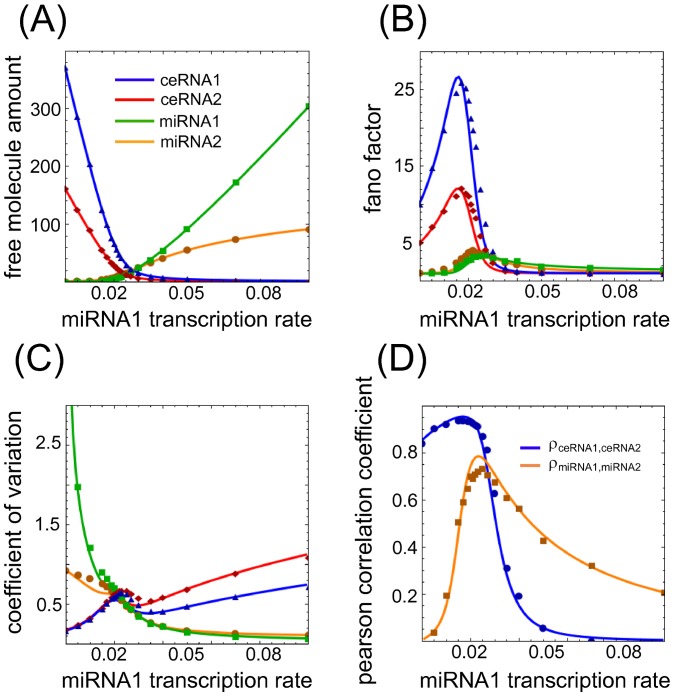
Threshold, noise and Pearson's coefficients varying miRNA transcription rate. (A–C) Steady state value for means, Fano factors and coefficients of variation for each free molecular species in a system of two miRNAs (miRNA1 and miRNA2, green and orange lines respectively) interacting with two ceRNAs (ceRNA1 and ceRNA2, blue and red lines respectively) varying the concentration of miRNA1. In proximity to the threshold the system shows hypersensitivity to changes in the control parameter (miRNA1 transcription rate), captured by a maximum in the Fano factors (panel B). For the same values of miRNA1 transcription rate, the local maximum in the coefficients of variation (panel C) is the fingerprint of bimodal distributions in the number of molecules for each molecular species. (D) Pearson's coefficients between the two miRNAs (orange line) and the two ceRNAs (blue line). The two lines show a maximum in proximity to the miRNA1 transcriptiom rate threshold value, meaning that there is a region of parameters where the fluctuations in the number of ceRNAs or miRNAs are highly correlated. Lines are the results of Gaussian approximation while symbols are Gillespie's simulations. For panels B,C the line color-code is the same as in panel A.

The onset of a threshold-like response as a consequence of a titration mechanism is a rather well known phenomenon [11], [13]–[18]. In Figures 2A and 3A, we show an example of threshold effect in the case 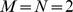 as a function of different ceRNA and miRNA concentrations. Such an effect can be derived under the assumption that the joint probability distributions of the different molecular species are statistically independent, as explained in Section Materials and Methods.

In a general network of interaction of 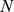 ceRNAs and 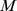 miRNAs, when miRNA-target interaction strength is high, following the derivation of Eq. 11 and depending on the control parameter we decide to tune, two distinct phases emerge: (i) if all target transcription rates are below the threshold level, explicitly computable in terms of all other model's parameters, all targets turn out to be bounded in complexes and the free molecule (*i.e.* not bounded) share is very low, (ii) if at least one of the transcription rates is above threshold, then all other target free molecule shares are expressed in finite amount. As shown in Figure 2A, the emerging scenario entails a cross-talk mechanism where a single mRNA target above threshold is able to drive the other common mRNA targets above threshold. The hypothesis of a strong ceRNA-miRNA interaction can be relaxed, and still, a smoother threshold-like behavior is observed [11].

One of the most controversial issue of the ceRNA hypothesis is to what extent can a 2–5 fold change in the abundance of one miRNA target (say ceRNA1), with realistic transcription rate and miRNA expression rate, affects hundreds of other targets of this same miRNA. To settle this controversy, in Table 1 we report the fold-change in the number of free ceRNAs and miRNA for a system of one miRNA interacting with 100 targets. We study which is the impact of the variation of a single ceRNA (ceRNA1 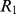) transcription rate (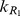) on another randomly chosen of the remaining 99 (let call it ceRNA2 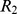). For simplicity (but this simplification can be relaxed), all the transcription, degradation and association rates are maintained equal among the different ceRNAs (these values are reported in File S1). Depending on the number of free miRNA S available, the system could be below, around or above the threshold, with consequently different miRNA dilution effects on the 100 miRNA targets. Although the fold-change in 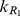 is the same in the three cases (we report the case of fold-change 1,5, and 7), the variation of ceRNA2 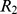 (and each one of the other 98 ceRNAs) and miRNA S levels are maximal when the system is in proximity to the threshold, while almost nothing changes when above or below the threshold. CeRNA1 grows almost linearly with its transcription rate below and above threshold, while again its variation is maximal in proximity to the threshold. Consider now the behavior of two different ceRNA networks characterized by the same transcription rates of the different chemical species (all ceRNAs have the same transcription rates across the two networks): (i) *network1* composed by 2 ceRNAs and 1 miRNA (N = 2,M = 1), (ii) *network2* analogous to the previously discussed case (N = 100, M = 1). It is now clear that if *network1* is at threshold, of course *network2* would be well above threshold (there would not be enough miRNAs), and conversely if *network2* is at threshold, *network1* would be well below threshold (there would be too many miRNAs and basically all ceRNAs would be bound by a miRNA). So the overall stoichiometry of the system dictates whether or not there is cross-talk between ceRNAs (see Table 1).

**Table 1:** Fold change in the number of free miRNA and ceRNAs.

above threshold: miRNA S transcription rate *k_S_ = *0.05*_S_* ^−1^(*k_S_ = *0.0001*S* ^−1^)			
*K_R_*_1_[*S* ^−1^]	*R*_1_ fold-change	*R*_2_ fold-change	*S* fold-change
0.1→0.2	2	1	1
0.1→0.5	5	1	1
0.1→0.7	7	1	1

near threshold: miRNA S transcription rate *k_S_ = *1.6*_S_* ^−1^(*k_S_ = *0.02*S* ^−1^)			
*K_R_*_1_[*S* ^−1^]	*R*_1_ fold-change	*R*_2_ fold-change	*S* fold-change
0.1→0.2	∼3(∼4)	∼1.3(∼2)	0.8(∼0.3)
0.1→0.5	15	3	∼0.4(∼0.1)
0.1→0.7	∼32(∼23)	4.5(∼3.3)	0.2(∼0.1)

below threshold: miRNA S transcription rate *k_S_ = *2*_S_* ^−1^(*k_S_ = *0.02*S* ^−1^)			
*K_R_*_1_[*S* ^−1^]	*R*_1_ fold-change	*R*_2_ fold-change	*S* fold-change
0.1→0.2	∼2	∼1	1(∼0.9)
0.1→0.5	∼5.5	∼1.1	∼0.9(∼0.8)
0.1→0.7	∼8.5(∼10.7)	∼1.2(∼1.5)	∼0.8(∼0.7)

Using ceRNA1 transcription rate 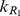 as control parameter we evaluate the fold change in the number of free miRNA (S), ceRNA1 (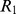) and ceRNA2 (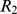) upon a variation of 2,5 and 7 fold in 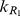 in a system of 1 miRNA interacting with 100 different targets (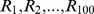). Depending on the availability of free miRNA S (which depends on its basal transcription rate 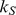) the system could be below, near or above the threshold. Although the fold-change in 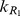 is the same in the three cases, the fold change in 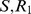 and 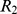 (measured as the ratio between their final and initial values) is maximal in proximity to the threshold. To obtain approximately the same fold-change in a system with only 1 miRNA and 2 targets (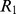 and 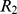) the miRNA transcription rate has to be lower. Its value is reported in brackets as well the corresponding fold-change when different from the case with 1 miRNA and 100 targets.

Interestingly enough we note that if, as control parameter, we decide to tune the p-th miRNA transcription rate, keeping all the remaining model's parameters fixed, a mirror-like scenario emerges (as displayed in Figure 3A): in complete analogy with the case previously discussed, also miRNAs cross-talk through ceRNAs. Here again, as long as all miRNAs transcription rates are below threshold, free miRNA molecule shares are very low. As the first miRNA transcription rate crosses the threshold, all other miRNAs show a substantial increase of their free share. In this case too there is a clear cross-talk between miRNAs. It is interesting to note that the threshold value predicted by the model (see Section Materials and methods) occurs at near-equimolar concentrations of the different chemical species.

If a hierarchy is present for the miRNA-target interaction strengths 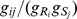 [11], [18], for example accounting for different MREs for different target mRNAs, then a hierarchy will be also established in the other target (miRNA) signal amplification levels when the amount of target mRNAs (miRNAs) is moved from below to above the threshold value. Targets sharing similar MREs will be more co-regulated than targets sharing only few MREs [18]. The miRNA-target interplay may thus be selective depending on the particular affinities and binding strengths [11], [12]. This leads to a complex regulatory network with non-trivial indirect interactions among targets in competition for the same pool of miRNAs.

**Figure 4:**
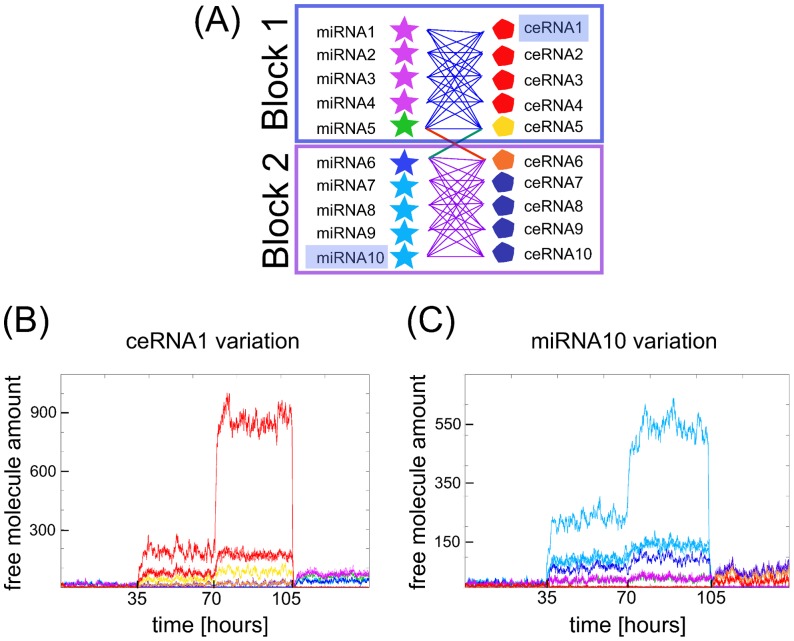
Selectivity of miRNA and ceRNA interactions. (A) Example of a network of ten miRNAs interacting with ten ceRNAs in blocks. The interaction links are such that we can define two main blocks (block 1 and block2) of strongly interacting miRNAs-ceRNAs connected by one common miRNAs (miRNA 5 in block 1, miRNA 6 in block 2) and ceRNAs (ceRNA 5 in block 1 and ceRNA 6 in block 2). Panels (B,C) show an example of dynamics of such network. Varying ceRNA1 (panel B) or miRNA10 (panel C) transcription rate during time (every 35 hours in the example, but the time is arbitrary) has a differentiated effect on the other ceRNAs and miRNAs present in the all network. The color-code for lines in panels B and C follows the color of miRNAs and ceRNAs in panel A.

The network sketched in Figure 1A is a crude simplification of what should be a real-case ceRNA's network. To make things slightly more realistic see Figure 4A, where two groups of ceRNAs interact through two distinct sets of miRNAs. However, a small subset of miRNAs makes the two groups of ceRNAs, otherwise statistically independent, *weakly interacting* by cross-connecting the two sets. We simulated the network's dynamics using the Gillespie algorithm in two different settings. In the first one, we modulate over time the transcription rate of one ceRNA, starting with a value below threshold, and we first increase the transcription of one specific ceRNA (ceRNA1) rate after 35 hours. A first observation is that it is enough to bring above threshold a single ceRNA to set the whole network in its non-repressed state. The second observation is that ceRNA-mediated regulation can be specific, *i.e.* we observe a clear hierarchy in the response of the different ceRNAs (see Figure 4B): those ceRNAs sharing the largest set of miRNA (red pentagons) respond more than the others. A second increase in the transcription rate of ceRNA1 after 70 hours makes the hierarchy in the responses even more clear. Interestingly, also the sets of ceRNAs (orange and blue pentagons) which do not share any targeting miRNA respond to the over-expression of ceRNA1 (although less than the other groups), thanks to an indirect effective interaction: ceRNA1 pulls up the red and yellow pentagon sets, the yellow pentagon pulls up the orange, and the latter the blue pentagon set.

In the second setting (see Figure 4C), we analyze the mirror scenario in which miRNA10 transcription rate is increased. Again the hierarchical responses of the different miRNAs is clearly visible.

### Beyond mean field approximation: noise and correlation coefficients

To get insight into molecular species correlations for the miRNA-ceRNA interaction network we then assume that the joint probability distribution 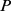 for the different molecular species is a multivariate Gaussian (see Section Materials and Methods). This *ansatz* turns out to be useful since all moments of a multivariate Gaussian can be expressed as a function of the first two, *i.e.* in terms of means and covariances. We will assume that the vector 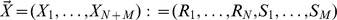 is distributed according a Gaussian multivariate measure of mean 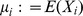 and covariances 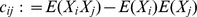. Thus the generic third and fourth moments read 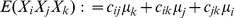 and 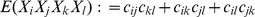.

In this way we are able to obtain a closed system of equations for 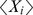, 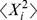 and 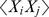 (see File S1 for a detailed analysis). This assumption is not arbitrary (the usual van Kampen's expansion method [31] shows the master equation is Gaussian except for small corrections) and interestingly performs better than the most widely used linear noise approximation (see File S1) when compared with Gillespie's simulations (see [32] for a nice introduction to the subject). Under this approximation we then find an analytical expression for means, noise and Pearson's correlation coefficients.

The threshold is characterized not only by the abrupt change of the mean quantities as a function of the control parameter, but also by Pearson's correlation coefficients and noise (both related to the covariances) which turn out to show a maximum around the threshold. For each molecular species we evaluated in terms of variance 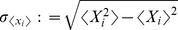 the Fano factor, 
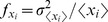
, and the coefficient of variation, 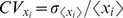, which are both measures of noise. While the first one tells how much a particular process is different from a Poisson process, the second is a dispersion index. Figures 2B,C and 3B,C show such noise profiles as a function of ceRNA1 or miRNA1 transcription rate. As it is possible to notice in Figures 2B and 3B, in proximity to the threshold the joint probability distributions are far from being independent (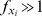 for all indexes 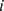 labelling the different chemical species) while a multivariate Gaussian approximation is better suited to describe the simulation results. In Figures 2C and 3C we plot the CV profiles. Increasing the ceRNA1 (miRNA1) transcription rate we observe a decreasing noise profile for ceRNAs (miRNAs) and an increasing one for miRNAs (ceRNAs), as expected because of the increasing and decreasing amount of free ceRNAs (miRNAs) and miRNAs (ceRNAs) respectively. Interestingly however, right close to the threshold it is possible to notice a bump in the CV profiles. This phenomenon, due to variances growing faster than means, is compatible with the bimodal distributions experimentally observed and verified via simulations in particular controlled conditions in bacterial sRNA target [33], [34].

The Pearson's correlation coefficients, 
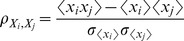
, are shown in Figures 2D and 3D. The profile of the curves as a function of the control parameter, with a well-defined maximum, confirms the system hypersensitivity near the threshold. Analogously, we can define the Pearson correlation coefficient between miRNAs and ceRNAs (not shown). In this case, miRNAs and ceRNAs are negatively correlated.

It is interesting to notice that exactly where the number of interacting molecules is small and the noise profiles show local maxima, the statistical correlation between molecular species is maximal too. Speculatively, the titration interaction mechanism provides for a tool able to affect the network homeostasis: potentially interacting ceRNAs (or miRNAs) needed in the same time fluctuate together.

### Threshold effect and miRNA-target catalytic interaction

So far we considered a titrative stoichiometric (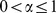) ceRNA/miRNA interaction. However, the open question is if cross-talk among miRNAs or miRNA targets can be possible in case of purely catalytic-like interaction (that is, in case of complete miRNA recycling, or rather 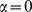 in Equation 1) [29].

It is straightforward to see that, at the steady state, equations for the various 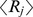 (or 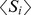) decouple when 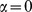 (see Equation 9) [18]. As a consequence, no cross-talk is possible among ceRNAs (or miRNAs). We found that in the out of equilibrium phase instead, the behavior is different.

**Figure 5:**
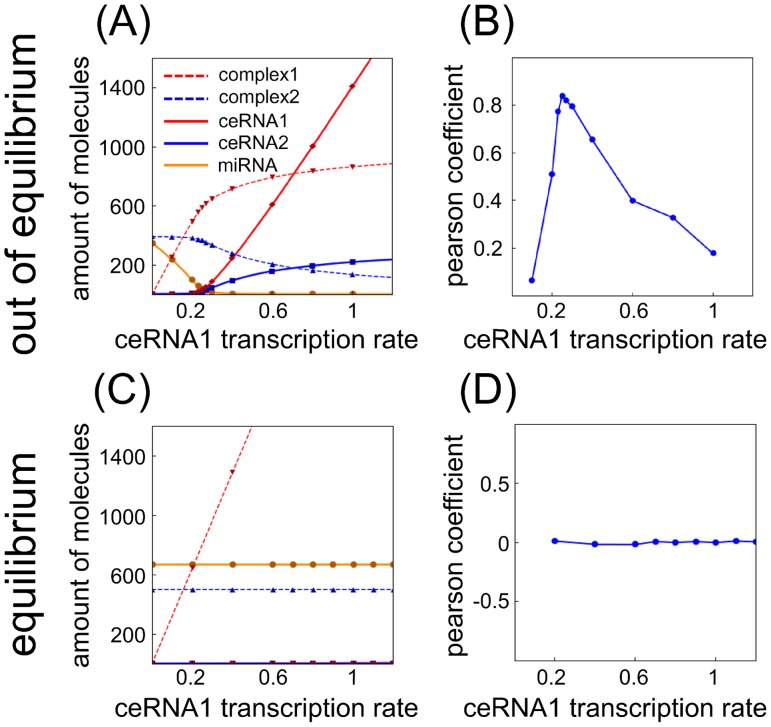
Threshold effect in a miRNA-target catalytic interaction. Example of a system of one miRNA interacting with two ceRNAs with cataliticity parameter 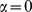. The threshold effect is possible only if the system is out of equilibrium (A). Numerical integration of Equation (1) in File S1 leads to time evolution of each molecular species for a given set of parameters. In panels A,C we plot "pictures" of the evolving system at different time 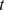 (panel A 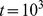, panel C 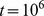) as a function of ceRNA1 transcription rate. When t is smaller than the time complexes need to reach their steady state a threshold effect is observed. In panels B,D we plot the corresponding Pearson's coefficient profiles. Where the threshold effect is present (panel A), a peak in the Pearson's coefficient is also observed.

We considered the time evolution of the system in Equation 1 of the Supplementary Material File S1, and then took pictures of the system at a given time 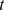. If 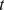 is sufficiently small with respect to the time the complexes need to reach the steady-state, for different values of miRNA (or ceRNA) transcription rate we can observe the threshold behavior of Figure 5A. Consequently ceRNAs or miRNAs cross-talk is possible, and statistical correlations are maximal, as shown by the Pearson's correlation coefficient profile in Figure 5B.

The emerging picture is that of a dynamical threshold whose value at a given time t tends monotonously to the equilibrium one in case of 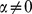 and to infinity in case of 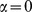 for large time. In the latter case no cross-talk is observed at equilibrium (Figure 5C,D).

The ceRNA effect is therefore robust also in case of catalytic miRNA-target interaction, the crucial point lieing in the instant of time at which we look at the system.

### Response times

We have already discussed the threshold effect due to titrative miRNA-target interaction and how the system displays strong sensitivity (maximum cross-talk) and maximal statistical correlation. We now want to understand how fast the system responds to an external perturbation. In particular we want to compute the time needed for a particular ceRNA (say ceRNA1) to reach the equilibrium after the instantaneous over-expression or knock-out of a second ceRNA (ceRNA2).

Following [35], we consider two different settings: (i) to mimic a sudden signal which saturates ceRNA2 promoter at 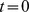, the transcription rate 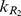 of ceRNA2 switches from zero to a given value (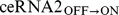), (ii) to mimic the opposite condition of a sudden drop of the activating signal at 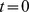, the transcription rate of ceRNA2 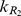 switches from its initial value to zero (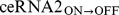).

Defining the response time as the time needed to reach half of the way between initial and final ceRNA1 steady state, we evaluate the response times for both switch-on (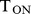) and switch-off (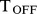) conditions (*i.e.* for 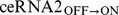 and 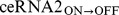 respectively). We integrated numerically the deterministic system of equations obtained with 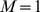 and 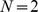 (see Equation 2 in Supplementary Material File S1) to calculate: (i) the time 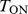 such that 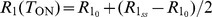 (where 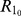 and 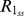 are the initial and final ceRNA1 steady-state respectively), (ii) the time 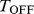 such that 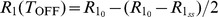. The initial conditions are 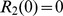 and 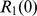 and 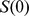 with their steady state values in absence of 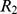 in the former case, and 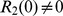 and 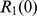 and 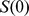 with their steady state values in presence of 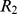 in the latter. We also considered a slightly more complex network in which more ceRNAs are present and we compute ceRNA1 response time with 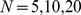.

We then ask two questions: (i) how the response time of ceRNA1 changes at different values of basal miRNA concentration, and (ii) what happens when the system is complicated by the addition of other competing targets.

**Figure 6:**
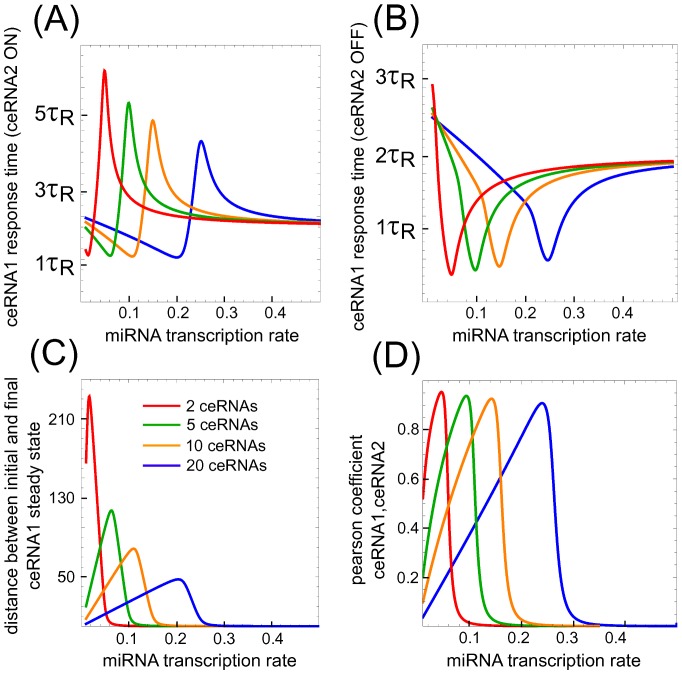
Response times upon one ceRNA perturbation. Increasing miRNA transcription rate ceRNA1 shows a maximum and a minimum in its response times upon switching on or off ceRNA2 transcription respectively (panel A and B). The maximum (minimum) is located near the threshold, where ceRNA1 initial value (that is its values before switching on (off) ceRNA2) is near to the steady state it will reach upon switching on (off) ceRNA2 (panel C) but is also more sensitive to ceRNA2 variation (look at the maximum in the Pearson's correlation coefficient between ceRNA1 and ceRNA2 in panel D). Different color lines correspond to different numbers of ceRNAs in competition for the same miRNA. The qualitative trend for response times and Pearson's correlation coefficient is robust with respect to increasing such number.

As displayed in Figure 6A,B, upon increasing miRNA transcription rate ceRNA1 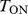 and 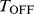 show a maximum and a minimum respectively. Both the maximum and the minimum are located at the threshold, where ceRNA1 initial and final equilibrium values are near (see Figure 6C). Such response time trend suggests an *out-of-equilibrium phase transition*, for which the system experiences anomalous dynamical features around threshold. Let us point out that around threshold, despite the change in terms of number of molecules from initial and final steady state is small, as depicted in Figure 6C, 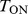 is largely increased while 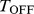 is decreased. Moreover, the qualitative shape of the curve is robust with respect to the number of targets in competition for the same miRNA (see Figure 6A,B where different line colors correspond to a different number of ceRNAs in the interaction's network): the maximum (resp. the minimum) of the response time depends only mildly on the number of ceRNA competitors, whereas the location of the threshold at which the free molecule share of ceRNA1 starts being repressed depends linearly on the number of competitors. Moreover, the statistical correlation between ceRNA1 and ceRNA2 seems independent from the size of the ceRNA's network: the maximum level of correlation is almost the same upon increasing the number of ceRNAs with only a shift to higher miRNA transcription rates (Figure 6D). Therefore ceRNA1 and ceRNA2 are always very correlated, notwithstanding the dynamical anomalies in the response-time around threshold.

### Network motifs and cross-talk

Which is the impact of recurrent wiring patterns on the general picture we analytically described, beyond the particular miRNA-target titrative interaction? It is increasingly clear that similarly to what happens in the transcriptional network [36], also in the mixed one (i.e. the superposition of transcriptional and post-transcriptional layers of regulation) network motifs can be detected [37]–[42]. The widespread idea is that motifs have been the object of selective pressure because of functional reasons, possibly playing elementary regulatory roles. As an example, miRNA-mediated feedback and feedforward loops have been found to be recurrent network motifs in mammals [39]. A recent work pointed out the function of incoherent miRNA-mediated feedforward loops (a transcription factor as master regulator of a miRNA and a target of both) in reducing noise from upstream regulators [43]. In [35] a minimal version of such motif (i.e. an intronic miRNA-mediated self regulation) has been mathematically analyzed. The results show that independently of the particular set of parameters considered the functions performed by the circuits are related with the maintenance of homeostasis. Moreover, in both these circuitries the analytical predictions are robust with respect to different modeling strategies for the miRNA-target interaction, including the titrative one. In this respect, the capability of the circuitries in reducing noise propagating from upstream regulators depends on the particular value of the 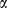 parameter.

While a complete mathematical characterization of those topologies from the point of view of crosstalk and correlation is out of the scope of the present work, we show through simulations that the threshold/cross-talk behavior of the system is maintained also in presence of feedbacks and feedforward loops (see Figure S4 in File S1). We thus compare the simplest system consisting of one miRNA and two ceRNAs with the cases in which (i) one of the two ceRNAs (ceRNA 1 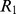) is translated and its protein product activates the miRNA transcription (feedback loop) and (ii) both the miRNA and one of the two ceRNAs are activated by a common transcription factor (incoherent feedforward loop). For cases (i) and (ii) we maintained fixed the parameters in common with the simplest circuit (one miRNA and two ceRNAs) while using reasonable translation and protein degradation rates. The transcription activation is modeled via a Hill function.

In Figure S4 in the File S1 we depicted the time evolution for free miRNA S and ceRNAs 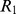 and 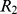 in the three cases (panels B,D,F). We chose as control parameter 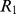 transcription rate and, as in Figure 4 of the main text, we increased its value from below to above threshold every 35 hours. As it is possible to notice, also in presence of feedback or feedforward loops it is enough to move one parameter (among those defining the threshold) to control the dynamical behavior of all the miRNA/ceRNAs players.

## Discussion

In this paper we analyzed the theoretical framework for the stochastic description of a general network of 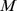 miRNAs interacting with 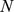 target mRNAs via a titration mechanism. With a dexterous use of the moment generating function approach plus simple Gaussian approximation we showed that it is possible to obtain analytical expressions for means and covariances for all the interacting molecules present in the system.

We have first shown how the already well understood threshold effect implied by titrative interaction [11], [13]–[16] entails with interesting cross-talk phenomena which, so far, have been only partially investigated from the experimental point of view [1]–[3], [8]–[10]. In particular the issue of the mirror scenario – for which not only ceRNAs cross-talk through competing for the same set of miRNAs, but, symmetrically the same set of miRNAs cross-talk through the common set of ceRNA – is a straightforward verification of the *ceRNA hypothesis* which, at the best of our knowledge, has never been attempted so far. In practice, knowing the set of miRNAs belonging to a specific ceRNA network, one could knock-down (resp. over-express) a given miRNA in the network. In this case, the model predicts that the other miRNAs in the network, driven by the controlled miRNA knock-down (resp. over-expression), should decrease (resp. increase) their free molecule share. Such an effect could be directly measurable as an up-regulation (resp. down-regulation) of any of the miRNAs targets (either belonging to the same ceRNA network, or to any other secondary target).

In addition to cross-talk and threshold phenomena, the model predicts interesting and experimentally measurable trends for the noise and Pearson's correlation coefficient profiles. In proximity to the threshold, where all the free molecular species involved in the system are present in small numbers, both the noise measures we analyzed (Fano factor and coefficient of variation) show a maximum (for the latter the maximum is local). These behaviors can be interpreted in terms of bimodal distributions for each molecular species involved in the titrative mechanism [34]. Interestingly the bimodality has been experimentally measured in a simple sRNA-mediated circuit in bacteria [33], and could be potentially verified in our ceRNA case.

In proximity to such threshold value, also the Pearson's correlation coefficients among ceRNAs or miRNAs show a maximum, meaning that the statistical correlation among molecules deriving from different genes is high. That is, not only the system is hypersensitive to little changes in the control parameter, but also fluctuations are highly correlated. As a matter of fact, the titration mechanism of interaction establishes a positive coupling among ceRNAs belonging to different genes (or among miRNAs). While the intensity of such correlation depends mostly on the combination of the basal transcription rates of each particular gene (so that different genes speak each other at different intensities, but the level of correlation is established by the particular parameters), the location of the maximum is determined by all the molecular species in competition. Furthermore, such statistical correlation is robust with respect to the number of ceRNAs involved in the system (with just a shift in the location of the threshold when increasing the number of ceRNAs) and also with respect to the catalyticity parameter 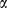. When 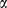 is zero, meaning that all the miRNAs are recycled, it is still possible to observe the threshold effect and the maximum in correlations' profiles as an out-of-equilibrium characteristic of the system. Thus, the *ceRNA effect* is always present, provided that the observation's time is short enough.

To investigate experimentally these features, quantitative fluorescence microscopy seems, for the time being, the most promising technique. Previous works not directly related to the *ceRNA hypothesis* (see [11] for a seminal work in bacteria, and [17] in human cell lines) used two-colors fluorescent reporter systems. The construct typically consists of a bidirectional drug-inducible promoter driving the expression of the two fluorescent proteins. The 3′UTR of the fluorescent proteins can be engineered to control the binding sites, and so the miRNA-mRNA binding affinity for the targeting miRNAs of interest. Both in [11] and [17], the method was used to monitor the threshold effect in a simple sRNA/miRNA 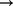 mRNA interaction. At the expenses of creating more complex constructs, an analogous technique could be deployed to investigate threshold, cross-talk, and noise/correlation behavior of simple ceRNA networks. In the most straightforward implementation one needs two reporter constructs: (i) the first construct consists of a bidirectional reporter system composed by the 3′UTR of ceRNA1 concatenated to the fluorescent gene (say green), and on the other side a miRNA binding site free 3′UTR concatenated to a second fluorescent gene (say yellow) to monitor the transcription activity, (ii) the second construct consists of a single reporter composed by the 3′UTR of ceRNA2 concatenated with a third fluorescent gene (say cherry). In this way one could simultaneously monitor the activity of both ceRNAs (green, cherry) as a function of the transcriptional activity of ceRNA1 (yellow) which would validate both qualitatively (in terms of the profile predicted by the model) and possibly quantitatively (by allowing a multi-parametric fit of the model's kinetic constants from the experimental data) the model predictions as displayed, for instance, in Figure 2.

Finally, the model shows interesting out-of-equilibrium features around threshold which could be experimentally testable (see Figure S3 in File S1). In particular the peculiar response time profile as a function of the distance from the threshold could be directly measured by means of quantitative time-lapse fluorescence microscopy [44] and flow cytometry to monitor ceRNAs dynamics. To monitor the dynamics of two ceRNAs, one could conservatively construct a two color fluorescent reporter system that allows for simultaneous monitoring of protein levels (see again[11], [17]). Of course larger networks could be potentially monitored using multiple colors.

Although a quantitative understanding of the impact of miRNA target cross-talk is still lacking, its mathematical characterization should specifically be addressed in the context of different regulatory conditions. For example, the presence of feedback or feedforward loops can confer peculiar features to a network, as the capability of enhancing or reducing noise at a particular node. However, threshold, cross-talk and increased correlation near the threshold seem to be a general characteristic due to the titrative miRNA/target interaction.

## Materials and Methods

### Stochastic simulations

Stochastic simulations have been performed via implementation of Gillespie's first reaction algorithm [45].

#### Theoretical framework: stochastic model

In analogy with Figure 1B, for each gene belonging to the miRNA-target network in Figure 1A we consider the key steps of transcription, degradation and titrative interaction among transcripts. Thus, the system is described by 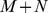 variables (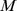 miRNAs 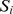 and 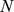 target mRNAs 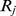 transcribed from 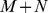 different genes) and the probability of finding in a cell exactly 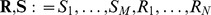 molecules at time 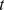 satisfies the following master equation:
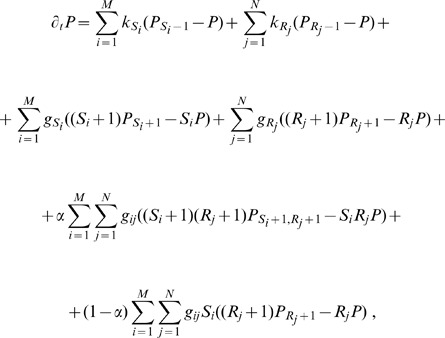
(1)with 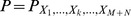 and 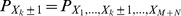. In Equation 1 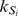 and 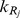 are transcription rates and 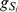 and 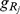 degradation rates for the 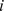-th miRNA and the 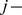
th target mRNA respectively. 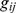 is the effective association rate for miRNA 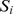 and its target 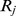. 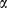 is the catalyticity parameter described above.

By defining the generating function,
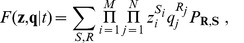
(2)where 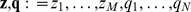, we can convert Equation 1 into the following second-order partial differential equation:
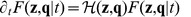
(3)where the operator 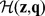 is defined as:



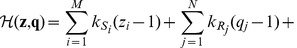
(4)

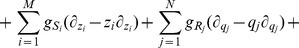


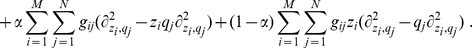



The moment generating function has the following properties:
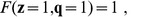
(5)

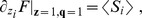


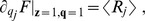


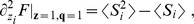


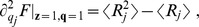


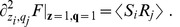
(6)


Considering higher order derivatives in Equation 3 at steady state (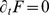), and assuming that all derivatives are computed in 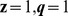, we find:
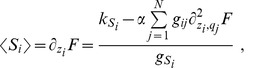
(7)

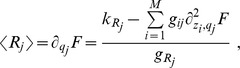


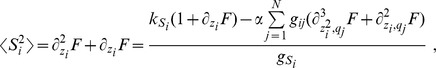


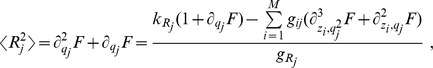


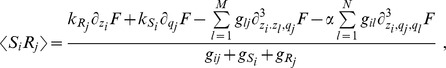


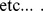



The moment-generating function defined in Equation 3 is unfortunately too complicated to be computed analytically even at steady state, as all moments depend on higher ones and the system is not closed, as shown in Equations 7. In the following we will present a series of increasingly accurate approximations for analyzing it.

### Independent molecular-species approximation

As a first step for determining analytically the behavior of the system, we will assume that the probability distribution 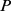 is factorized:
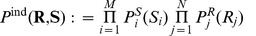
(8)


Under this assumption it turns out that the steady state solution for the 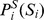, and 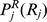 are Poisson distributions whose mean value can be expressed solving the following second order system of equations,
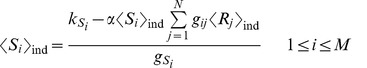
(9)

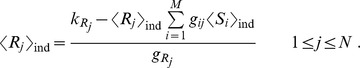



Analytic solutions for the system of Equations 9 can be easily written in the case 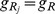, 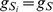 and 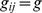 for all 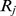 and 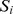:
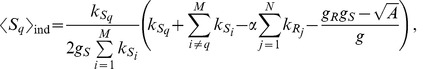
(10)

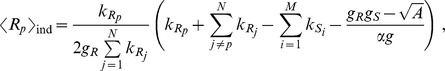
with 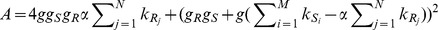. In the more general and biologically relevant case of different molecules half-lives and complex affinities 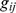, solutions can still be found, but they turn out to be too complex and long to be reported here.

### Locating the threshold

The simplest way to locate the threshold is to solve the system of Equations 9 in the limit of strong miRNA-target interaction (high 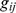) thus finding:
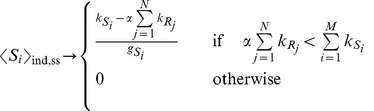
(11)

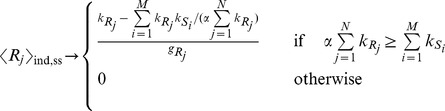



The threshold position is determined by the relative amount of miRNAs and their targets (see Equation 11). For fixed 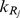 and 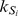, with 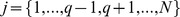 and 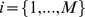, the threshold is set by 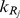 and by all miRNA transcription rates 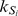. Thus, as long as the q-th mRNA target transcription rate 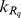 is below its threshold level 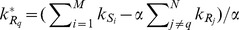 all targets are bound in complexes and their free molecule amount is very low (while miRNAs are expressed), or, in other terms, the threshold is located at near-equimolar concentration of the different chemical species.

In principle there is no reason for the parameter 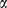 to be the same for all the miRNAs. As a first approximation we could assume that 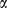 is only target dependent. The result will be a shift in the threshold position (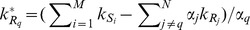). Unfortunately no explicit form exists for the general case where 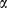 depends both on targets and miRNAs (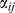). Given the poor knowledge of the specific value for 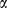, in the following we will assume a constant value for it.

Increasing 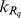 beyond its threshold results in the expression of all the other targets (while miRNAs will be all bound in complexes), see Figure 2A.

Within the independent chemical species approximation in Equation 8 the Fano factor (noise index 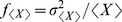) for each molecular species is 1. The factorized approximation is good enough in showing the threshold effect, but fails in determining correlations among molecular species (see symbols, which are the results of Gillespie's simulations, in Figures 2A and 3A).

### Gaussian Approximation

The simplest approximation beyond mean-field is a Gaussian one. We denote 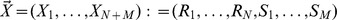. The approximation assumes that 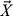 is distributed as a multivariate Gaussian:
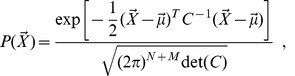
(12)where the covariance matrix 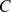 has coordinates 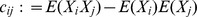, the vector 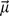 has coordinates 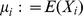, and the expectation value 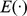 is with respect to the Gaussian measure 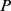 defined in Equation 12. All moments of a Gaussian multivariate measure can be expressed in terms of 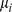 and 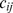. Therefore the moments derived from the generating function in Equation 7 can be expressed in terms of 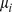 and 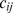. In the Supplementary Material File S1 we describe in details the computation of the specific 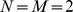 case, and we compare the performance of the Gaussian approximation with the linear-noise approximation.

## Supporting Information

File S1 **Supporting information.**
(PDF)
