# Supplementary material for: Modelling Competing Endogenous RNA Networks
Source: PLoS One. 2013 Jun 26;8(6):e66609. doi: 10.1371/journal.pone.0066609 (PMC3694070; doi:10.1371/journal.pone.0066609)
Supplement: File S1 — Supporting information. (PDF) [file pone.0066609.s001.pdf]

Modelling competing endogenous RNA networks - Supporting Information

Carla Bosia[1*], Andrea Pagnani[1,2], Riccardo Zecchina[1,2]
1 Human Genetics Foundation (HuGeF), Via Nizza 52, I-10126, Torino, Italy.
2 Physics Department and Center for Computational Sciences, Politecnico Torino, Corso Duca degli Abruzzi 24, I-10129, Torino, Italy.
† These authors contributed equally to this work.
∗ E-mail: carla.bosia@hugef-torino.org

Contents

1 Generalized mean-field equation with explicit complexes 2

2 Generalized master equation with explicit complexes 3

3 Gaussian Approximation 3

4 Linear noise approximation 5

5 Figure and table parameters (main text) 8

6 Response times and experimentally testable trend 10

7 Network motifs and cross-talk 10

1 Generalized mean-field equation with explicit complexes

We describe the general case of N different target mRNAs interacting with M different miRNAs. The action of a miRNA on its target has the following characteristics: each miRNA molecule can constitute a complex with a target molecule and then can be eventually released. The molecular species are: free miRNAs (S_i), free mRNAs (R_j), complexes C_{ij} of miRNA S_i with mRNA R_j. Each gene can be transcribed with rate $k_{\{R_i,S_j\}}$ and degraded with rate $g_{\{R_i,S_j\}}$. Complexes C_{ij} associate with rate k_{ij}^+ and dissociate with rate k_{ij}^-. Each complex eventually degrade with rate γ_{ij}. A schema of such network is represented in Figure S1. The mean-field equations thus read:

$$\begin{aligned}
\frac{dR_j}{dt} &= k_{R_j} - g_{R_j}R_j + \sum_{i=1}^{M}\left(-k_{ij}^+ S_i R_j + k_{ij}^- C_{ij}\right) \\
\frac{dS_i}{dt} &= k_{S_i} - g_{S_i}S_i + \sum_{j=1}^{N}\left(-k_{ij}^+ S_i R_j + k_{ij}^- C_{ij} + (1-\alpha)\gamma_{ij}C_{ij}\right) \\
\frac{dC_{ij}}{dt} &= k_{ij}^+ R_j S_i - (k_{ij}^- + \gamma_{ij})C_{ij} \qquad (1)
\end{aligned}$$

with $i \in \{1,\ldots,M\}$ and $j \in \{1,\ldots,N\}$. Assuming that complexes reach the equilibrium faster than the other molecular species, we can simplify the system of Equations (1) to the following one:

$$\begin{aligned}
\frac{dS_i}{dt} &= k_{S_i} - g_{S_i}S_i - \alpha\sum_{j=1}^{N} g_{ij}S_iR_j \qquad (2) \\
\frac{dR_j}{dt} &= k_{R_j} - g_{R_j}R_j - \sum_{i=1}^{M} g_{ij}S_iR_j\ ,
\end{aligned}$$

with $g_{ij} = \frac{k_{ij}^+\gamma_{ij}}{k_{ij}^- + \gamma_{ij}}$.

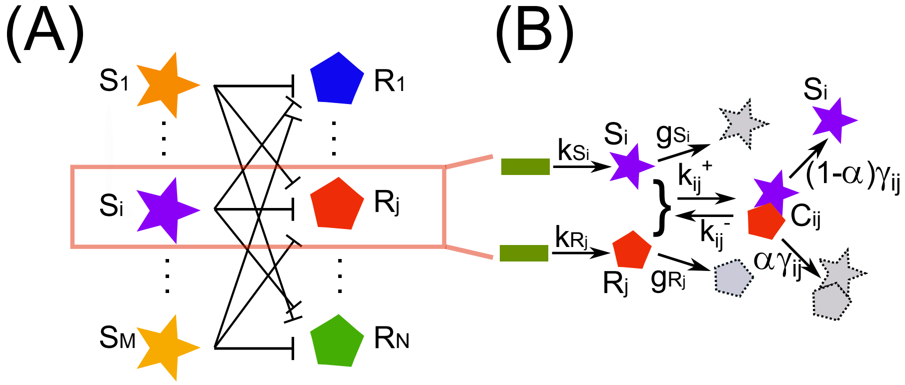

Figure S1. *Representation of a generic miRNA-target interaction network.* (A) Simplified picture of a miRNA-ceRNA interaction network. (B) For each miRNA (S_i) and ceRNA (R_j) present in the network we consider the main steps of transcription (rates k_{S_i} and k_{R_j} respectively) and degradation (rates g_{S_i} and g_{R_j} respectively) plus a titrative interaction between miRNA and ceRNA. miRNA and ceRNA can therefore form a complex C_{ij} with association rate $k^+_{ij}$and dissociation rate k^-_{ij}. The complex can then degrade with rate γ_{ij}. The parameter α (the catalyticity parameter) tells which is the probability that a miRNA is recycled after interacting with one of its targets.

2 Generalized master equation with explicit complexes

The master equation corresponding to Equation 1 reads:

$$\begin{aligned}\partial_t P &= \sum_{i=1}^{M} k_{S_i}(P_{S_i-1,R_j,C_{ij}} - P_{S_i,R_j,C_{ij}}) + \sum_{j=1}^{N} k_{R_j}(P_{S_i,R_j-1,C_{ij}} - P_{S_i,R_j,C_{ij}}) + \\ &+ \sum_{i=1}^{M} g_{S_i}((S_i+1)P_{S_i+1,R_j,C_{ij}} - S_i P_{S_i,R_j,C_{ij}}) + \sum_{j=1}^{N} g_{R_j}((R_j+1)P_{S_i,R_j+1,C_{ij}} - R_j P_{S_i,R_j,C_{ij}}) + \\ &+ \sum_{i=1}^{M}\sum_{j=1}^{N} k^+_{ij}((S_i+1)(R_j+1)P_{S_i+1,R_j+1,C_{ij}-1} - S_i R_j P_{S_i,R_j,C_{ij}}) + \\ &+ \sum_{i=1}^{M}\sum_{j=1}^{N} k^-_{ij}((C_{ij}+1)P_{S_i-1,R_j-1,C_{ij}+1} - C_{ij} P_{S_i,R_j,C_{ij}}) + \\ &+ \alpha \sum_{i=1}^{M}\sum_{j=1}^{N} \gamma_{ij}((C_{ij}+1)P_{S_i,R_j,C_{ij}+1} - C_{ij} P_{S_i,R_j,C_{ij}}) + \\ &+ (1-\alpha) \sum_{i=1}^{M}\sum_{j=1}^{N} \gamma_{ij}((C_{ij}+1)P_{S_i-1,R_j,C_{ij}+1} - C_{ij} P_{S_i,R_j,C_{ij}}) \,,\end{aligned} \tag{3}$$

3 Gaussian Approximation

We work here in some details the explicit computation for the Gaussian approximation in the specific case of 2 microRNAs (S_1, S_2) and 2 ceRNAs (R_1, R_2). Denoting with $\partial^{l+m}_{z_i^l,q_j^m} F := \partial^{l+m}_{z_i^l,q_j^m} F|_{\mathbf{z},\mathbf{q}=(1,\ldots,1)}$, at

steady state the system of equation reads:

$$
\begin{aligned}
\partial_{z_1}F &= \frac{k_{z_1}-\alpha g(\partial^2_{z_1,q_1}F+\partial^2_{z_1,q_2}F)}{g_S}\\
\partial_{z_2}F &= \frac{k_{z_2}-\alpha g(\partial^2_{z_2,q_1}F+\partial^2_{z_2,q_2}F)}{g_S}\\
\partial_{q_1}F &= \frac{k_{q_1}-g(\partial^2_{z_1,q_1}F+\partial^2_{z_2,q_1}F)}{g_R}\\
\partial_{q_2}F &= \frac{k_{q_2}-g(\partial^2_{z_1,q_2}F+\partial^2_{z_2,q_2}F)}{g_R}\\
\partial^2_{z_1^2}F &= \frac{k_{z_1}\partial_{z_1}F-\alpha g(\partial^3_{z_1^2,q_1}F+\partial^3_{z_1^2,q_2}F)}{g_S}\\
\partial^2_{z_1,z_2}F &= \frac{k_{z_2}\partial_{z_1}F+k_{z_1}\partial_{z_2}F-2\alpha g(\partial^3_{z_1,z_2,q_1}F+\partial^3_{z_1,z_2,q_2}F)}{2g_S}\\
\partial^2_{z_1,q_1}F &= \frac{k_{q_1}\partial_{z_1}F+k_{z_1}\partial_{q_1}F-g(\partial^3_{z_1^2,q_1}F+\partial^3_{z_1,z_2,q_1}F+\alpha\partial^3_{z_1,q_1^2}F+\alpha\partial^3_{z_2,q_1,q_2}F)}{g+g_R+g_S}\\
\partial^2_{z_1,q_2}F &= \frac{k_{q_2}\partial_{z_1}F+k_{z_1}\partial_{q_2}F-g(\partial^3_{z_1^2,q_2}F+\partial^3_{z_1,z_2,q_2}F+\alpha\partial^3_{z_1,q_1,q_2}F+\partial^3_{z_2,R_2^2}F)}{g+g_R+g_S}\\
\partial^2_{z_2^2}F &= \frac{k_{z_2}\partial_{z_2}F-\alpha g(\partial^3_{z_2^2,q_1}F+\partial^3_{z_2^2,q_2}F)}{g_S}\\
\partial^2_{z_2,q_1}F &= \frac{k_{q_1}\partial_{z_2}F+k_{z_2}\partial_{q_1}F-g(\partial^3_{z_1,z_2,q_1}F+\partial^3_{z_2^2,q_1}F+\alpha\partial^3_{z_2,q_1^2}F+\alpha\partial^3_{z_2,q_1,q_2}F)}{g+g_R+g_S}\\
\partial^2_{z_2,q_2}F &= \frac{k_{q_2}\partial_{z_2}F+k_{z_2}\partial_{q_2}F-g(\partial^3_{z_1,z_2,q_1}F+\partial^3_{z_2^2,q_1}F+\alpha\partial^3_{z_2,q_1^2}F+\alpha\partial^3_{z_2,q_1,q_2}F)}{g+g_R+g_S}\\
\partial^2_{q_1^2}F &= \frac{k_{q_1}\partial_{q_1}F-g(\partial^3_{z_1,q_1^2}F+\partial^3_{z_2,q_1^1}F)}{g_R}\\
\partial^2_{q_1,q_2}F &= \frac{k_{q_2}\partial_{q_1}F+k_{q_1}\partial_{q_2}F-2g(\partial^3_{z_1,q_1,q_2}F+\partial^3_{z_2,q_1,q_2}F)}{g+g_R+g_S}\\
\partial^2_{q_2^2}F &= \frac{k_{q_2}\partial_{q_2}F-g(\partial^3_{z_1,q_2^2}F+\partial^3_{z_2,q_2^2}F)}{g_R}
\end{aligned}
\tag{4}
$$

Recalling that within the Gaussian approximation the partial derivatives of the third order can be

expressed in terms of that of lower order:

$$
\begin{aligned}
\partial^3_{z_1^2,z_2}F &= (\partial^2_{z_1^2}F+\partial_{z_1}F)\partial_{z_2}F+2\partial_{z_1}F\partial^2_{z_1,z_2}F-2(\partial_{z_1}F)^2\partial_{z_2}F-\partial^2_{z_1,z_2}F\\
\partial^3_{z_1^2,q_1}F &= (\partial^2_{z_1^2}F+\partial_{z_1}F)\partial_{q_1}F+2\partial_{z_1}F\partial^2_{z_1,q_1}F-2(\partial_{z_1}F)^2\partial_{q_1}F-\partial^2_{z_1,q_1}F\\
\partial^3_{z_1^2,q_2}F &= (\partial^2_{z_1^2}F+\partial_{z_1}F)\partial_{q_2}F+2\partial_{z_1}F\partial^2_{z_1,q_2}F-2(\partial_{z_1}F)^2\partial_{q_2}F-\partial^2_{z_1,q_2}F\\
\partial^3_{z_1,z_2^2}F &= (\partial^2_{z_2^2}F+\partial_{z_2}F)\partial_{z_1}F+2\partial_{z_2}F\partial^2_{z_1,z_2}F-2(\partial_{z_2}F)^2\partial_{z_1}F-\partial^2_{z_1,z_2}F\\
\partial^3_{z_1,q_1^2}F &= (\partial^2_{q_1^2}F+\partial_{q_1}F)\partial_{z_1}F+2\partial_{q_1}F\partial^2_{z_1,q_1}F-2(\partial_{q_1}F)^2\partial_{z_1}F-\partial^2_{z_1,q_1}F\\
\partial^3_{z_1,q_2^2}F &= (\partial^2_{q_2^2}F+\partial_{q_2}F)\partial_{z_1}F+2\partial_{q_2}F\partial^2_{z_1,q_2}F-2(\partial_{q_2}F)^2\partial_{z_1}F-\partial^2_{z_1,q_2}F\\
\partial^3_{z_2^2,q_1}F &= (\partial^2_{z_2^2}F+\partial_{z_2}F)\partial_{q_1}F+2\partial_{z_2}F\partial^2_{z_2,q_1}F-2(\partial_{z_2}F)^2\partial_{z_1}F-\partial^2_{z_1,q_1}F\\
\partial^3_{z_2^2,q_2}F &= (\partial^2_{z_2^2}F+\partial_{z_2}F)\partial_{q_2}F+2\partial_{z_2}F\partial^2_{z_2,q_2}F-2(\partial_{z_2}F)^2\partial_{z_1}F-\partial^2_{z_1,q_2}F\\
\partial^3_{z_2,q_1^2}F &= (\partial^2_{q_1^2}F+\partial_{q_1}F)\partial_{z_2}F+2\partial_{q_1}F\partial^2_{z_2,q_1}F-2(\partial_{q_1}F)^2\partial_{z_2}F-\partial^2_{z_2,q_1}F\\
\partial^3_{z_2,q_2^2}F &= (\partial^2_{q_2^2}F+\partial_{q_2}F)\partial_{z_2}F+2\partial_{q_2}F\partial^2_{z_2,q_2}F-2(\partial_{q_2}F)^2\partial_{z_2}F-\partial^2_{z_2,q_2}F\\
\partial^3_{z_1,z_2,q_1}F &= \partial^2_{z_1,z_2}\partial_{q_1}F+\partial^2_{z_2,q_1}\partial_{z_1}F+\partial^2_{z_1,q_1}\partial_{z_2}F-2\partial_{z_1}F\partial_{z_2}F\partial_{q_1}F\\
\partial^3_{z_1,z_2,q_2}F &= \partial^2_{z_1,z_2}\partial_{q_2}F+\partial^2_{z_2,q_2}\partial_{z_1}F+\partial^2_{z_1,q_2}\partial_{z_2}F-2\partial_{z_1}F\partial_{z_2}F\partial_{q_2}F\\
\partial^3_{z_2,q_1,q_2}F &= \partial^2_{z_2,q_1}\partial_{q_2}F+\partial^2_{z_2,q_2}\partial_{q_1}F+\partial^2_{q_1,q_2}\partial_{z_2}F-2\partial_{z_2}F\partial_{q_1}F\partial_{q_2}F\\
\partial^3_{z_1,q_1,q_2}F &= \partial^2_{z_1,q_1}\partial_{q_2}F+\partial^2_{z_1,q_2}\partial_{q_1}F+\partial^2_{q_1,q_1}\partial_{z_1}F-2\partial_{z_1}F\partial_{q_1}F\partial_{q_2}F
\end{aligned}
\tag{5}
$$

Inserting relations (5) into (4) we obtain a closed system of 14 equations in 14 unknowns. In the general case of a network of N ceRNAs interacting through M miRNAs we would have a complete system of $2(N+M)+\binom{N+M}{2}$ equations.

4 Linear noise approximation

We use the linear noise approximation [1] in order to obtain the steady state fluctuation covariance matrix directly from the macroscopic system. For a general system of M miRNAs interacting with N mRNAs and R elementary reactions, we assign to each reaction r a propensity f_r defined from the probability $\Omega f_r(\psi,\Omega)\delta t$ that a reaction r occurs in the homogeneous system volume Ω in the time interval δt. ψ is the concentration vector of the $M+N$ chemical components of the system. In the macroscopic limit ($\Omega\to\infty$) the system dynamics is described by the following $M+N$ ordinary differential equations,

$$\frac{d\psi_p}{dt} = \sum_r \nu_{rp} f_r(\psi_1,...,\psi_{M+N}) \,, \tag{6}$$

where ν_{rp} is the $rp-$th element of the stoichiometry matrix, i.e. it indicates the number of molecules by which a component p changes when an elementary reaction of type r occurs.

For small enough deviations $\delta\psi=[\delta\psi_1,\delta\psi_2,...,\delta\psi_{M+N}]$ from its steady state, the dynamics of Equation (6) can be approximated by a system of linear differential equations, according to $\frac{\delta\psi}{dt}=\mathcal{A}\delta\psi$, where $\mathcal{A}$ is the Jacobian matrix with elements

$$a_{pq} = \sum_{r=1}^{R}\nu_{rp}(\frac{\partial f_r}{\partial\psi_q})_{\psi_{ss}} \,. \tag{7}$$

The master equation for the probability of having $X=[X_1,X_2,...,X_p,...,X_{M+N}]$ molecules in the

system at time t is then

$$\frac{dP}{dt}(X,t) \quad = \quad \Omega\sum_{r=1}^{R}(\prod_{p=1}^{N}E_p^{\nu_{rp}}-1)f_r(X\Omega^{-1},\Omega)P(X,t)\ , \tag{8}$$

with E being a step operator with property $E_p^{\nu_{rp}}g(...,X_p,...)=g(...,X_p+\nu_{rp},...)$.

To obtain the linear noise approximation [1] we expand the master equation to second order in $\Omega^{-1/2}$ after substituting each p-th component of X with $X_p=\Omega\psi_p+\Omega^{1/2}x_p$. x_p is the p-th component of a new random vector x such that the X_p is thus described as a macroscopic term $\Omega\psi_p$ plus a stochastic term $\Omega^{1/2}x_p$. We thus obtain a linear Fokker-Planck equation for the joint probability distribution $\Pi(x,t)$ of x:

$$\frac{d\Pi}{dt}(x,t) \quad = \quad -\sum_{p,q}a_{pq}\frac{\partial x_p\Pi}{\partial x_p}+\frac{1}{2}\sum_{p,q}b_{pq}\frac{\partial^2\Pi}{\partial x_p x_q}\ . \tag{9}$$

The matrix elements a_{pq} are given by the Jacobian matrix $\mathcal{A}$, while the elements b_{pq} of the diffusion matrix $\mathcal{B}$ are defined as in [2],

$$b_{pq} \quad = \quad \sum_{r=1}^{R}f_r\nu_{rp}\nu_{pq}\ . \tag{10}$$

Generally $\mathcal{A}$ and $\mathcal{B}$ may depend on time, but here we will restrict our analysis to the steady state case. In this way, the stationary solution of Equation (9) is the normal distribution $N(0,\Xi)$. Ξ, which is the covariance matrix with elements ξ_{rp}, is the solution of the matrix Lyapunov equation:

$$\mathcal{A}\Xi+\Xi\mathcal{A}^T+\mathcal{B}=0 \tag{11}$$

The covariance matrix $\mathcal{C}$ for the deviations in molecule number (δX_i) is related to Ξ via the relation $\mathcal{C}=\Omega\Xi$. Thus, in the linear noise approach the expected value $\langle X_r\rangle$ is approximated by $\Omega\psi_r$ and the true covariance σ_{rp}^2 by c_{rp}. Then, the expressions for Pearson's correlation coefficients (ρ_{X_r,X_p}), Fano factors (f_X) and coefficients of variation (CV_X) can be easily derived:

$$\begin{aligned}
\rho_{X_r,X_p} &= \frac{\sigma_{rp}^2}{\sigma_{rr}\sigma_{pp}}\sim\frac{c_{rp}}{\sqrt{c_{rr}c_{pp}}}=\frac{\xi_{rp}}{\sqrt{\xi_{rr}\xi_{pp}}}\ ,\\
CV_{X_r} &= \frac{\sigma_{rr}}{\langle X_r\rangle}\sim\frac{\sqrt{c_{rr}}}{\Omega\psi_r}=\frac{\sqrt{\xi_{rr}}}{\psi_r}\ ,\\
f_{X_r} &= \frac{\sigma_{rr}^2}{\langle X_r\rangle}\sim\frac{c_{rr}}{\Omega\psi_r}=\frac{\xi_{rr}}{\psi_r}\ .
\end{aligned} \tag{12}$$

Therefore, thanks to Equation (11) the matrix $\mathcal{C}$ (and thus the stochastic properties of a system) can be directly evaluated from macroscopic parameters.

Let's now discuss in details the specific case with two ceRNAs in interaction with one miRNAs. In such a system, the propensity vector f assumes the following form:

$$f:=\{k_{S_1},S_1g_{S_1},g_{11}S_1R_1,g_{12}S_1R_2,k_{S_2},S_2g_{S_2},g_{21}S_2R_1,g_{22}S_2R_2,k_{R_1},R_1g_{R_1},k_{R_2},R_2g_{R_2}\}\ , \tag{13}$$

and the stoichiometry matrix ν is given by:

$$\nu = \begin{pmatrix} 1 & 0 & 0 & 0 \\ -1 & 0 & 0 & 0 \\ -\alpha & 0 & -1 & 0 \\ -\alpha & 0 & 0 & -1 \\ 0 & 1 & 0 & 0 \\ 0 & -1 & 0 & 0 \\ 0 & -\alpha & -1 & 0 \\ 0 & -\alpha & 0 & -1 \\ 0 & 0 & 1 & 0 \\ 0 & 0 & -1 & 0 \\ 0 & 0 & 0 & 1 \\ 0 & 0 & 0 & -1 \end{pmatrix}. \tag{14}$$

Thus, the Jacobian and diffusion matrices ($\mathcal{A}$ and $\mathcal{B}$ respectively) follow,

$$\begin{aligned} \mathcal{A} &= \begin{pmatrix} -g_{S_1} - \alpha(g_{11}R_1 + g_{12}R_2) & 0 & -\alpha g_{11}S_1 & -\alpha g_{12}S_1 \\ 0 & -g_{S_2} - \alpha(g_{21}R_1 + g_{22}R_2) & -\alpha g_{21}S_2 & -\alpha g_{22}S_2 \\ -g_{11}R_1 & -g_{21}R_1 & -g_R - g_{11}S_1 - g_{21}S_2 & 0 \\ -g_{12}R_2 & -g_{22}R_2 & 0 & -g_R - g_{12}S_1 - g_{22}S_2 \end{pmatrix} \\ \mathcal{B} &= \begin{pmatrix} k_{S_1} + g_{S_1}S_1 + \alpha^2 S_1 A & 0 & \alpha g_{11}R_1S_1 & \alpha g_{12}R_2S_1 \\ 0 & k_{S_2} + g_{S_2}S_2 + \alpha^2 S_2 B & \alpha g_{21}R_1S_2 & \alpha g_{22}R_2S_2 \\ \alpha g_{11}R_1S_1 & \alpha g_{21}R_1S_2 & k_{R_1} + R_1 C & 0 \\ \alpha g_{12}R_2S_1 & g_{22}R_2S_2 & 0 & k_{R_2} + R_2 D \end{pmatrix}, \end{aligned} \tag{15}$$

with $A = g_{11}R_1 + g_{12}R_2$, $B = g_{21}R_1 + g_{22}R_2$, $C = g_{R_1} + g_{11}S_1 + g_{21}S_2$ and $D = g_{R_2} + g_{12}S_1 + g_{22}S_2$. The covariance matrix elements c_{rp} can be evaluted accordingly. In Figure S2 we plot the Pearson correlation coefficient of such system as a function of ceRNA1 transcription rate. As it is possible to notice, Gaussian approximation performs better than Linear Noise approximation [3].

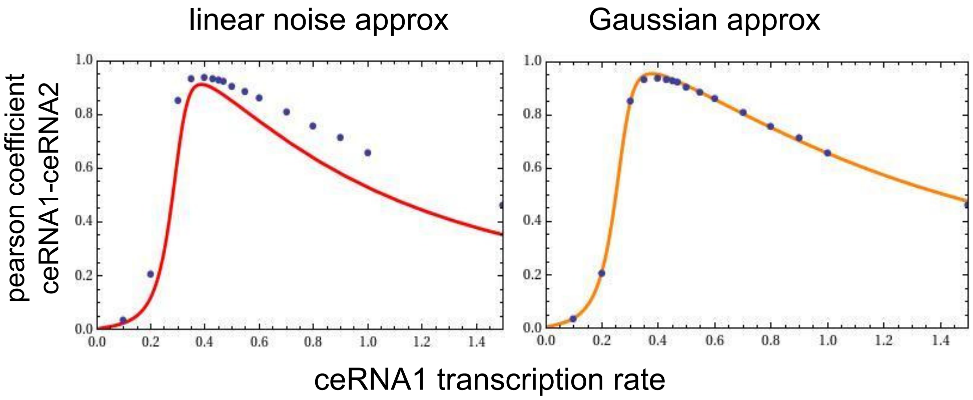

Figure S2. *Comparison between Linear Noise and Gaussian approximations.* (Left panel) Linear noise approximation, (Right panel) Gaussian approximation. Lines are analytical approximations of the Pearson correlation coefficient. Dots are the results of 10^4 Gillespie's simulations.

5 Figure and table parameters (main text)

Figure 2

miRNAs transcription rates: $k_{S_1} = 0.05s^{-1}$ and $k_{S_2} = 0.045s^{-1}$;
ceRNA2 transcription rate: $k_{R_2} = 0.155s^{-1}$;
miRNA degradation rates: $g_{S_1} = g_{S_2} = 0.0002s^{-1}$;
ceRNAs degradation rates: $g_{R_1} = g_{R_2} = 0.0004s^{-1}$;
ceRNA-miRNA association rates: $g_{11} = g_{12} = g_{21} = g_{22} = 0.0005s^{-1}$;
catalyticity parameter: $\alpha = 0.1$;
ceRNA1 transcription rate is the control parameter and ranges from 0 to $1.4s^{-1}$.

Figure 3

miRNA2 transcription rate: $k_{S_2} = 0.03s^{-1}$;
ceRNAs transcription rates: $k_{R_1} = 0.355s^{-1}$ and $k_{R_2} = 0.155s^{-1}$;
miRNA degradation rates: $g_{S_1} = g_{S_2} = 0.0002s^{-1}$;
ceRNAs degradation rates: $g_{R_1} = g_{R_2} = 0.0004s^{-1}$;
ceRNA-miRNA association rates: $g_{11} = g_{12} = g_{21} = g_{22} = 0.0005s^{-1}$;
catalyticity parameter: $\alpha = 0.1$;
miRNA1 transcription rate is the control parameter and ranges from 0 to $0.1s^{-1}$.

Figure 4

Panel (B):
miRNA1-10 transcription rates: $k_S = 0.075 + 0.01\text{rand}()s^{-1}$;
ceRNA2-10 transcription rate: $k_R = 0.15 + 0.01\text{rand}()s^{-1}$;
miRNA1-10 degradation rates: $g_S = 0.0002s^{-1}$;
ceRNAs1-10 degradation rates: $g_R = 0.0004s^{-1}$;
miRNA-ceRNA association rates: $g = 0.0006s^{-1}$
catalyticity parameter: $\alpha = 0.5$;
ceRNA1 transcription rate is the control parameter and every 35 hours takes the following values: $0.15s^{-1}$, $0.4s^{-1}$, $0.8s^{-1}$, $0s^{-1}$.

Panel (C):
miRNA1-9 transcription rate: $k_S = 0.075s^{-1} + 0.01\text{rand}()s^{-1}$;
ceRNA1-10 transcription rates: $k_R = 0.15 + 0.01\text{rand}()s^{-1}$;
miRNA1-10 degradation rates: $g_S = 0.0002s^{-1}$;
ceRNA1-10s degradation rates: $g_R = 0.0004s^{-1}$;
miRNA-ceRNA association rates: $g = 0.0006s^{-1}$;
catalyticity parameter: $\alpha = 0.5$;
miRNA1 transcription rate is the control parameter and every 35 hours takes the following values: $0.075s^{-1}$, $0.2s^{-1}$, $0.3s^{-1}$, $0s^{-1}$.

Figure 5

miRNAs transcription rates: $k_S = 0.2s^{-1}$;
ceRNA2 transcription rate: $k_{R_2} = 0.155s^{-1}$;
miRNA degradation rates: $g_{S_1} = g_{S_2} = 0.0003s^{-1}$;

ceRNAs degradation rates: $g_{R_1} = g_{R_2} = 0.0004s^{-1}$;
complex association rates: $k_1^+ = k_2^+ = 0.0005s^{-1}$;
complex dissociation rates: $k_1^- = k_2^- = 0.0003s^{-1}$;
complex degradation rates: $\gamma_1 = \gamma_2 = 0.00031s^{-1}$;
catalyticity parameter: $\alpha = 0.1$;
ceRNA1 transcription rate is the control parameter and ranges from 0 to $1.2s^{-1}$.

Figure 6

ceRNA1 transcription rates: $k_{R_1} = 0.155s^{-1}$;
ceRNA2$_{OFF \to ON}$ transcription rate jumps from $k_{R_2} = 0$ to $k_{R_2} = 0.345s^{-1}$;
ceRNA2$_{ON \to OFF}$ transcription rate jumps from $k_{R_2} = 0.345s^{-1}$ to $k_{R_2} = 0$;
miRNA degradation rates: $g_{S_1} = g_{S_2} = 0.0002s^{-1}$;
ceRNAs degradation rates: $g_{R_1} = g_{R_2} = 0.0004s^{-1}$;
ceRNA-miRNA association rates: $g_{11} = g_{12} = g_{21} = g_{22} = 0.0005s^{-1}$;
catalyticity parameter: $\alpha = 0.1$;
miRNA1 transcription rate is the control parameter and ranges from 0 to $0.5s^{-1}$. All the other ceRNAs have transcription rates $k_R = 0.1s^{-1}$ and all the other rates equal to ceRNA1 ones.

Table 1

ceRNA2 transcription rate: $0.15s^{-1}$;
ceRNAs from 3 to 100 ($R_3, ..., R_{100}$) transcription rates: $0.15s^{-1}$;
ceRNA degradation rates: $0.0004s^{-1}$;
miRNA transcription rate in case of 100 targets ($R_1, ..., R_{100}$): $0.05s^{-1} \to$ above threshold; $1.6s^{-1} \to$ near threshold; $2s^{-1} \to$ below threshold;
miRNA transcription rate in case of 2 targets (R_1, R_2): $0.0001s^{-1} \to$ above threshold; $0.02s^{-1} \to$ near threshold; $0.2s^{-1} \to$ below threshold;
miRNA degradation rate: $0.0002s^{-1}$;
catalyticity parameter: $\alpha = 0.1$;
ceRNA-miRNA association rates: $0.0005s^{-1}$;
ceRNA1 transcription rate k_{R_1} is the control parameter and takes the value $0.1, 0.5, 0.7s^{-1}$.

6 Response times and experimentally testable trend

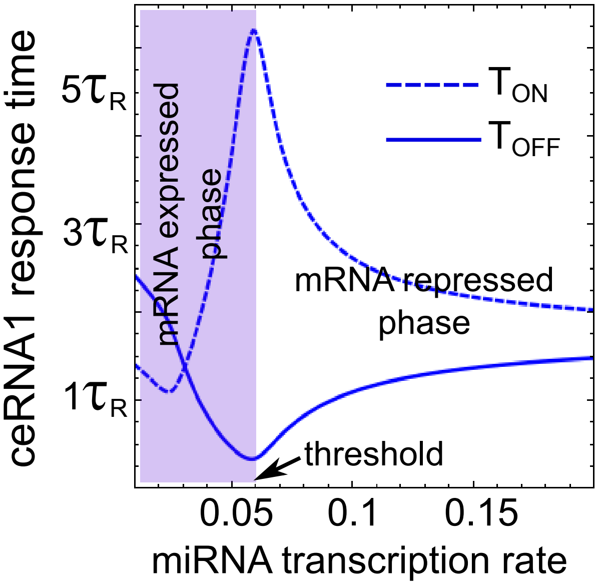

Figure S3. *Response times and experimentally accessible parameter region.* We show together the T_{ON} and T_{OFF} response times as present in Figure 6 in the main text for the case with 3 ceRNAs. The highlighted region corresponds to the experimentally accessible one. Increasing miRNA concentration, switch-off response times show a decreasing trend while switch-on a U-shaped one. The parameter setting is the same of Figure 6 (main text).

7 Network motifs and cross-talk

We analyzed three different network topologies in order to show that threshold and cross-talk are robust and maintained also in presence of feedback or feedforward loops. In Figure 4 (panel A) we schematically depict a simple system consisting of one miRNA S (cyan star) and two ceRNAs R_1 (red pentagon) and R_2 (blue pentagon). We then add other regulatory links to this circuit. In particular, in panel C ceRNA1 R_1 (red pentagon) is translated into protein P_1 (red circles) which in turn activates the miRNA (cyan star) transcription. The miRNA transcription rate k_S thus depends on the amount of protein P_1 via a Hill function: $k_S(P_1) = k_S^{max} P_1^c/(h^c + P_1^c)$. The circuit is now a feedback loop in which the miRNA has two targets R_1 and R_2 (blue pentagon) and one of them is the transcript of a transcription factor. In panel E instead both miRNA (cyan star) and ceRNA1 (red pentagon) are activated by a common transcription factor P (orange circles) which is in turn translated from mRNA R (orange pentagon). In this case, both S and R_1 transcription rates are dependent on the amount of P via the following Hill functions: $k_S(P) = k_S^{max} P^c/(h^c + P^c)$ and $k_{R_1}(P) = k_{R_1}^{max} P^c/(h^c + P^c)$. This circuit is thus an incoherent feedforward loop (as that analyzed in detail in [4]) in which the miRNA has two different targets R_1 and R_2 (blue pentagon). Every molecular species can degrade (grey broken figures) with constant rates and the interaction miRNA/target is modeled via a titration mechanism as that presented in the main text (and simplified in Figure 4 via violet lines in panel A,C,E). In panel B,D,F we show the dynamics for the amount of free miRNA S (cyan line) and ceRNAs R_1 (red line) and R_2 (blue line) for the three circuits when varying ceRNA1 transcription rate k_{R_1} ($k_{R_1}^{max}$ in the feedforward loop case). As in Figure 4

of the main text, we varied such parameter every 35 hours. The figure shows that also in case of different regulatory conditions it is possible to observe a threshold-like behavior: it is enough to tune only one parameter (k_{R_1}) to move both ceRNAs from below (for time $t < 35$ hours) to above (for time $t > 35$ hours) the threshold.
The parameters for the simulations in Figure 4 are the following:

Panel (B): *two ceRNA system*
ceRNA2 transcription rate: $k_{R_2} = 0.155s^{-1}$;
ceRNA degradation rates: $g_R = 0.0004s^{-1}$;
miRNA transcription rate: $k_S = 0.05s^{-1}$;
miRNA degradation rate: $g_S = 0.0002s^{-1}$;
catalyticity parameter: $\alpha = 0.1$;
ceRNA-miRNA association rates: $g = 0.0005s^{-1}$;
ceRNA1 transcription rate k_{R_1} is the control parameter and every 35 hours takes the following values: $0.15s^{-1}$, $0.4s^{-1}$, $0.8s^{-1}$.

Panel (D): *feedback loop*
ceRNA2 transcription rate: $k_{R_2} = 0.155s^{-1}$;
ceRNA degradation rates: $g_R = 0.0004s^{-1}$;
miRNA transcription rate: $k_S^{max} = 0.05s^{-1}$;
miRNA degradation rate: $g_S = 0.0002s^{-1}$;
catalyticity parameter: $\alpha = 0.1$;
ceRNA-miRNA association rates: $g = 0.0005s^{-1}$;
ceRNA1 translation rate: $k_{P_1} = 0.05s^{-1}$;
protein degradation rate: $g_{P_1} = 0.0002s^{-1}$;
Hill coefficient: $h = 10$;
Hill exponent: $c = 1$;
ceRNA1 transcription rate k_{R_1} is the control parameter and every 35 hours takes the following values: $0.15s^{-1}$, $0.4s^{-1}$, $0.8s^{-1}$.

Panel (E): *feedforward loop*
ceRNA2 transcription rate: $k_{R_2} = 0.155s^{-1}$;
ceRNA degradation rates: $g_R = 0.0004s^{-1}$;
miRNA transcription rate: $k_S^{max} = 0.05s^{-1}$;
miRNA degradation rate: $g_S = 0.0002s^{-1}$;
catalyticity parameter: $\alpha = 0.1$;
ceRNA-miRNA association rates: $g = 0.0005s^{-1}$;
ceRNA1 translation rate: $k_P = 0.05s^{-1}$;
protein degradation rate: $g_P = 0.0002s^{-1}$;
Hill coefficient: $h = 10$;
Hill exponent: $c = 1$;
ceRNA1 transcription rate $k_{R_1}^{max}$ is the control parameter and every 35 hours takes the following values: $0.15s^{-1}$, $0.4s^{-1}$, $0.8s^{-1}$.

References

1. van Kampen N (2007) Stochastic processes in physics and chemistry. North holland.

2. Risken H (1996) The Fokker-Planck equation: Methods of solution and applications, volume 18. Springer Verlag.

3. Lafuerza LF (2009) Gaussian approximation to the resolution of master equations. Master's thesis, Universitat de les Illes Balears.

4. Osella M, Bosia C, Corá D, Caselle M (2011) The role of incoherent microrna-mediated feedforward loops in noise buffering. PLoS Comput Biol 7: e1001101.

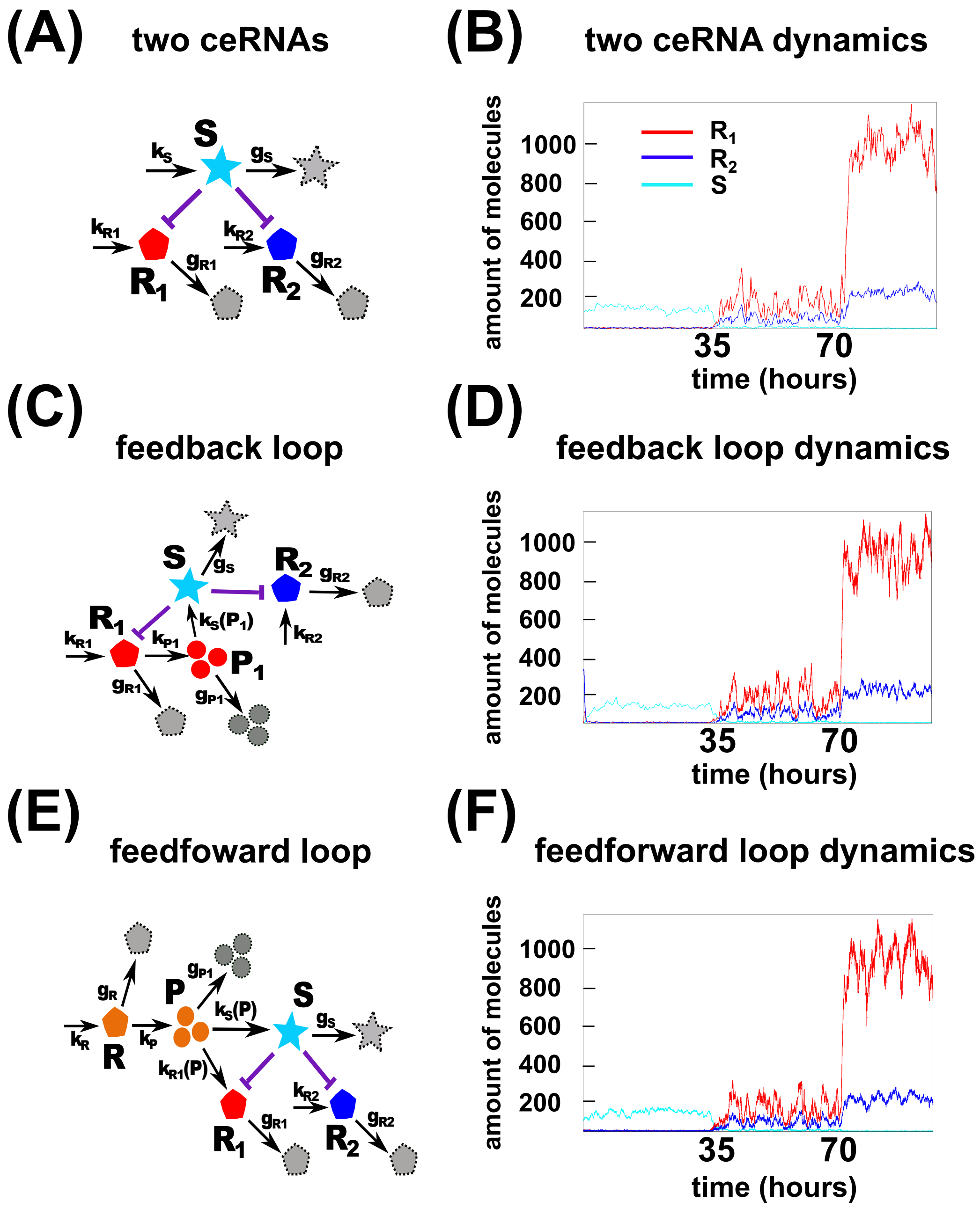

Figure S4. *Network motifs and cross-talk.* Example of dynamics for one miRNA S (cyan star) and two ceRNAs R_1 (red pentagon) and R_2 (blue pentagon) for three different topologies: (A) the simplest miRNA/ceRNAs motifs, (C) a feedback loop between miRNA and the protein translated from ceRNA1 P_1 (red circles) and (E) an incoherent feedforward loop for miRNA and ceRNA1 (which are thus activated by a common transcription factor P (orange circles)). In all those panels broken figures represent degraded molecules. The transcriptional activation due to the presence of a transcription factor (protein P_1 in (C) and protein P (E)) is modeled via a Hill function. Thus, in (C) $k_S(P_1) = (k_S^{max} \times P_1^c)/(h^c + P_1^c)$ while in (E) $k_S(P) = (k_S^{max} \times P^c)/(h^c + P^c)$ and $k_{R_1}(P) = (k_{R_1}^{max} \times P^c)/(h^c + P^c)$. Panel B,D,F show the dynamical behavior for the amount of free miRNA and ceRNAs for the three circuits. The parameters for panels B,D,F corresponding to reaction's rate in panels A,C,E are reported in Section 7.
